# Immunogenicity of plant‐produced African horse sickness virus‐like particles: implications for a novel vaccine

**DOI:** 10.1111/pbi.12783

**Published:** 2017-08-01

**Authors:** Susan J. Dennis, Ann E. Meyers, Alan J. Guthrie, Inga I. Hitzeroth, Edward P. Rybicki

**Affiliations:** ^1^ Biopharming Research Unit Department of Molecular and Cell Biology University of Cape Town Cape Town South Africa; ^2^ Equine Research Centre University of Pretoria Onderstepoort South Africa; ^3^ Institute of Infectious Disease and Molecular Medicine Faculty of Health Sciences University of Cape Town Cape Town South Africa

**Keywords:** virus‐like particle, African horse sickness virus, biopharming, transient expression, vaccines

## Abstract

African horse sickness (AHS) is a debilitating and often fatal viral disease affecting horses in much of Africa, caused by the dsRNA orbivirus African horse sickness virus (AHSV). Vaccination remains the single most effective weapon in combatting AHS, as there is no treatment for the disease apart from good animal husbandry. However, the only commercially available vaccine is a live‐attenuated version of the virus (LAV). The threat of outbreaks of the disease outside its endemic region and the fact that the LAV is not licensed for use elsewhere in the world, have spurred attempts to develop an alternative safer, yet cost‐effective recombinant vaccine. Here, we report the plant‐based production of a virus‐like particle (VLP) AHSV serotype five candidate vaccine by *Agrobacterium tumefaciens*‐mediated transient expression of all four capsid proteins in *Nicotiana benthamiana* using the cowpea mosaic virus‐based *HyperTrans* (CPMV‐*HT*) and associated pEAQ plant expression vector system. The production process is fast and simple, scalable, economically viable, and most importantly, guinea pig antiserum raised against the vaccine was shown to neutralize live virus in cell‐based assays. To our knowledge, this is the first report of AHSV VLPs produced in plants, which has important implications for the containment of, and fight against the spread of, this deadly disease.

## Introduction

African horse sickness (AHS) is a devastating illness of horses, which is frequently fatal in susceptible hosts, and has been part of South Africa's veterinary disease landscape for several centuries. It is widely recognized as one of the most lethal viral diseases of horses worldwide (Coetzer and Guthrie, 2004; Weyer *et al*., 2013). The disease is caused by a number of distinct serotypes of African horse sickness virus (AHSV), a group of nonenveloped isometric dsRNA viruses (genus *Orbivirus*, family Reoviridae) which are transmitted by biting midges of the *Culicoides* genus. AHS is infectious but noncontagious, and is endemic to sub‐Saharan Africa (Mellor and Hamblin, 2004; Sanchez‐Vizcaino, 2004). South Africa is one of the few countries where all nine serotypes of the virus have been isolated (von Teichman *et al*., 2010). However, the virus has occasionally escaped its geographical limitation and extended further afield to countries in North Africa, the Middle East, the Arabian Peninsula and the Mediterranean region (MacLachlan and Guthrie, 2010). Global climate change is thought to be contributing to the gradual northward migration of the midge vector, which has led to a sobering international awareness that AHS‐free countries with milder climate conditions are possibly increasingly at risk for outbreaks of the disease or even the establishment of endemicity (Herholz *et al*., 2008; Hopley and Toth, 2013; de Vos *et al*., 2012). The emergence of the generically related bluetongue virus (BTV) in north‐western Europe in 2006 (Darpel *et al*., 2007), as well as the extended AHS outbreak that occurred in western Mediterranean countries between 1987 and 1991 (Rodriguez *et al*., 1992), has only served to reinforce these concerns.

Disease control in South Africa has largely been effected by immunization with live‐attenuated vaccines (LAVs) produced by Onderstepoort Biological Products, based on products developed in the 1930s (Alexander, 1934). The currently used LAV is supplied in two polyvalent vials containing three and four AHSV serotypes each, but neither AHSV‐5 nor AHSV‐9 is included in the vaccine (von Teichman and Smit, 2008). Although the LAV is currently the best option in the fight against AHS, its use has raised concerns with regard to reversion to virulence, gene segment reassortment between outbreak and vaccine strains (Weyer *et al*., 2016), and the absence of DIVA, which is the ability to Differentiate between Infected and Vaccinated Animals. Most importantly, the LAV is not licensed for use outside of the African subcontinent.

As is presently the case in South and southern Africa, AHS outbreaks in Europe would result in large economic losses to the equine industry and would have an enormous emotional impact on owners and lovers of horses. There is thus a pressing need to develop new, safe, efficacious and cost‐effective DIVA vaccines which would primarily address the concerns of the South African equestrian community, as well as being acceptable prophylactic or rapid response vaccines in the European and other emerging outbreak contexts.

**Figure 1:**
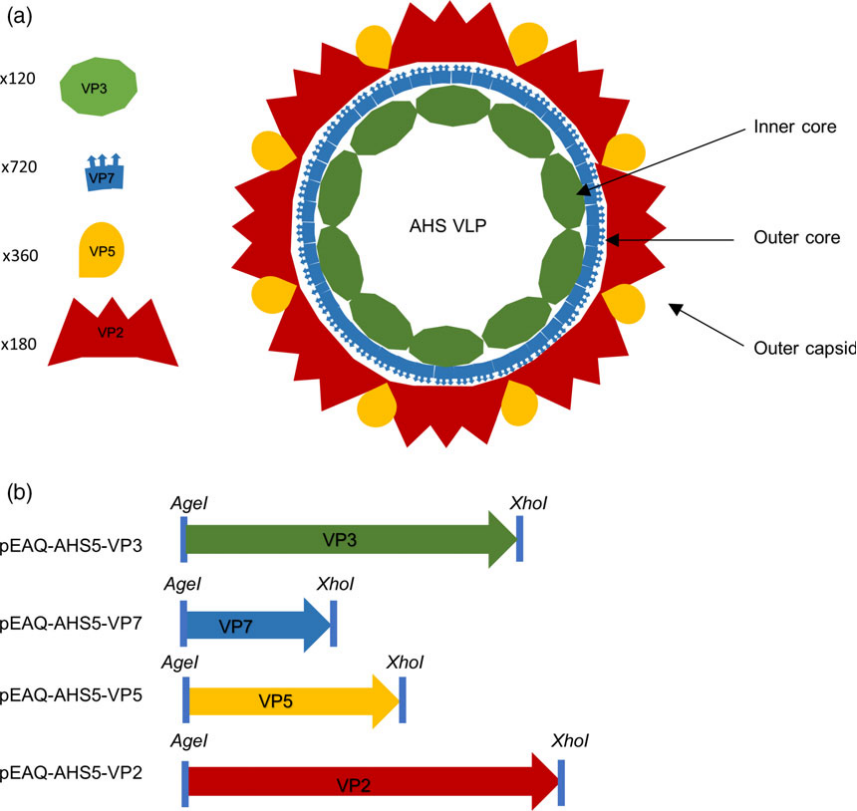
Schematic representation of the constructs created for *Agrobacterium‐*mediated expression of African horse sickness (AHSV) serotype 5 structural proteins in *N. benthamiana* and their resultant assembly into virus‐like particles. (a) Stoichiometric diagram of virus‐like particle formation. (b) Codon‐optimized genes for AHSV‐5 VP2, VP3, VP5 and VP7 were cloned into the pEAQ‐*HT* plant expression vector (Sainsbury *et al*., 2009).

The AHSV genome consists of 10 segments of linear double‐stranded RNA, encoding seven structural and five nonstructural proteins (Roy *et al*., 1994). The virion is nonenveloped and is composed of three distinct protein layers (Figure 1a). The inner core is formed by VP3, while the outer core layer is formed by protein VP7, which is also the group‐specific antigen used in ELISA‐based diagnostic tests (Chuma *et al*., 1992). VP7 assembles into trimers that attach perpendicularly to the VP3 surface. In native virions, these two layers enclose the subcore, comprising the 10 dsRNA segments together with the transcription complex, to form a stable icosahedral core particle around 78 nm in diameter. The outer capsid is composed firstly of a layer of VP5 and then one of VP2, which is the protein containing the antigenic determinants that induce serotype‐specific neutralizing antibodies.

Due to raised international awareness and local dissatisfaction with the current vaccine, AHSV research has focussed in recent years on the development of recombinant vaccines based on selected antigenic AHSV proteins, particularly the outer capsid proteins VP2 and VP5. Baculovirus expression systems (Kanai *et al*., 2014; Roy and Sutton, 1998) and poxvirus vectors (Alberca *et al*., 2014; Calvo‐Pinilla *et al*., 2014, 2015; El Garch *et al*., 2012; Guthrie *et al*., 2009) have been used to produce vaccines that induce protective immunity against various AHSV antigens. Disadvantages inherent to these types of vaccines include firstly, that recombinant soluble antigens are generally poorly immunogenic and require potent adjuvants or repeated boost inoculations to enhance immunogenicity; secondly, that pre‐existing immunity against the viral vector may compromise vaccine efficacy. Virus‐like particles (VLPs) that mimic the structure of intact virions, on the other hand, provide an attractive alternative vaccine platform. Sharing certain key characteristics with live viruses, VLPs are safe nonreplicating protein assemblies with the advantage of being highly immunogenic, as epitopes are displayed in ordered repetitive arrays on the particle surface (Noad and Roy, 2003). Such vaccines present no risk of reversion to virulence nor of dsRNA segment reassortment with wild virus strains because they do not contain viral RNA or nonstructural proteins, which also makes it possible to distinguish between vaccinated and infected animals using molecular diagnostic techniques.

In addition to expressing the individual AHSV VP2 and VP5 proteins, Roy and Sutton (1998) also used the baculovirus expression system in insect cells to synthesize AHS virus‐like particles. More recently, reverse genetics systems have been used to generate replication‐deficient AHSVs (Lulla *et al*., 2016; Vermaak *et al*., 2015; van de Water *et al*., 2015), which have the potential to be used as Disabled Infectious Single Animal (DISA) vaccine strains. Although this technology looks promising, the associated cost and upscaling requirements have thus far prevented any of these potential vaccine candidates from being commercialized.

Over recent years, the use of plant systems to express recombinant viral structural proteins, with the resulting self‐assembly of VLPs, has become increasingly popular as the method is both cost‐effective and free from the risk of contaminating animal pathogens (Lomonossoff and D'Aoust, 2016; Rybicki, 2010, 2014; Steele *et al*., 2017; Topp *et al*., 2016). Thuenemann *et al*. (2013) recently reported the high‐level expression of fully assembled VLPs of bluetongue virus serotype 8 (BTV‐8) in *N. benthamiana* using the pEAQ‐*HT* plant transient expression vector system (Sainsbury *et al*., 2009). The VLPs elicited a strong antibody response in sheep which provided protective immunity against challenge with a BTV‐8 field isolate. Furthermore, Medicago Inc. has reported the transient expression of rotavirus VLPs composed of four different capsid proteins in *N. benthamiana* (D'Aoust *et al*., 2013). Following on from these studies, here we report the expression and complete assembly of AHSV serotype 5 VLPs in *N. benthamiana* using *Agrobacterium*‐mediated delivery of constructs encoding the four major structural proteins of one of the AHSV serotypes not currently included in the LAV, serotype 5.

## Results

### AHSV‐5 capsid proteins transiently expressed in *N. benthamiana* leaves self‐assemble into VLPs

**Figure 2:**
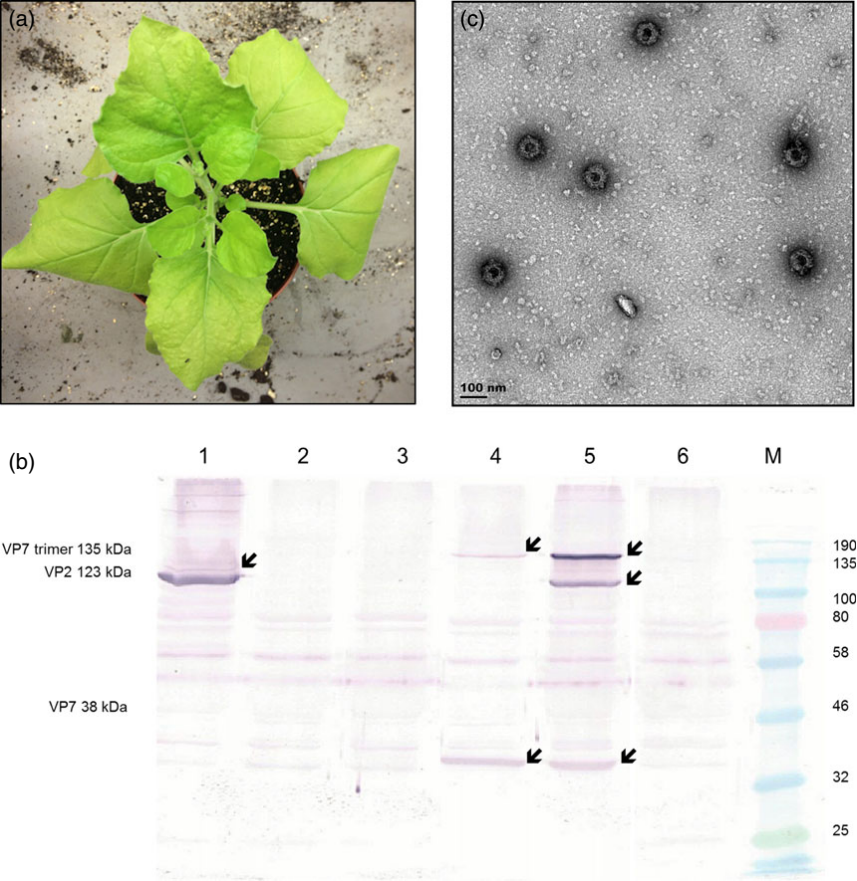
Expression of recombinant AHSV‐5 structural proteins and their assembly into virus‐like particles in *N. benthamiana*. (a) *N. benthamiana* plant 7 dpi with all four AHSV‐5 *Agrobacterium* recombinants. (b) Western blot analysis of crude leaf extracts obtained 7 dpi with *Agrobacterium radiobacter AGL1–ATCC BAA‐101* containing pEAQ‐AHS5 VP2 (lane 1), pEAQ‐AHS5 VP3 (lane 2), pEAQ‐AHS5 VP5 (lane 3), pEAQ‐AHS5 VP7 (lane 4) or co‐infiltrated with all four AHSV‐5 recombinants (lane 5). Crude extract from leaves infiltrated with *Agrobacterium* transformed with pEAQ‐*HT* expression vector lacking any gene of interest was used as a negative control (lane 6) on the same blot. Anti‐AHSV 5 antiserum (1 : 1000), which was unable to detect either VP3 or VP5, was used as the primary antibody. VP7 trimeric proteins (135 kDa), VP2 (123 kDa) and VP7 monomeric proteins (38 kDa) are indicated by arrow heads. Colour‐prestained protein standard, broad range (New England Biolabs, Massachusetts, USA) indicated to the right of the blot was used as a molecular weight marker. (c) Fully assembled AHSV 5 virus‐like particles imaged by TEM analysis of crude extracts from plants co‐infiltrated with pEAQ‐AHS5 VP2, pEAQ‐AHS5 VP3, pEAQ‐AHS5 VP5 and pEAQ‐AHS5 VP7. Scale bar, 100 nm.

To investigate whether AHSV‐5 capsid proteins could be transiently co‐expressed and lead to spontaneous self‐assembly of intact VLPs within individual plants, recombinant plasmids containing the VP2, VP3, VP5 and VP7 genes were constructed. A consensus sequence of each gene was obtained by aligning all the known sequences listed in GenBank; these were codon‐optimized for *Nicotiana* spp. translation and synthesized with flanking *AgeI* and *XhoI* restriction enzyme sites by GenScript Biotech Corporation, China. The genes were cloned into the multiple cloning site of the pEAQ‐*HT* vector (Sainsbury *et al*., 2009; obtained from G. Lomonossoff, John Innes Centre, UK) to yield four different constructs, pEAQ‐AHS5‐VP2, pEAQ‐AHS5‐VP3, pEAQ‐AHS5‐VP5 and pEAQ‐AHS5‐VP7 (Figure 1b). Transient expression of the AHSV proteins in *N. benthamiana* was tested by small‐scale syringe infiltration of five leaves per experiment with *Agrobacterium* strains carrying individual constructs, or co‐infiltration of the same plant with all four recombinant‐carrying strains. All infiltrated leaf tissue exhibited chlorosis, but little if any necrosis was observed (Figure 2a). *Agrobacterium* suspensions carrying recombinants in two different VP2 : VP3 : VP5 : VP7 ratios were tested, namely 1 : 1 : 1 : 1 and 1 : 1 : 2 : 1, as the latter ratio has been previously shown (van Zyl *et al*., 2016) to give a better yield of bluetongue virus (BTV) VLPs. Three leaf discs were clipped per expression test using an Eppendorf vial lid, and extracted on 3, 5 and 7 days postinfiltration (dpi), to determine the optimal expression conditions. Western blots showed that crude leaf extracts infiltrated with *Agrobacterium‐*carrying recombinants at an OD_600_ of 0.5 each and prepared 7 days after infiltration yielded good protein expression (Figure 2b). There was no apparent difference in expression between the two construct mixture ratios used (Figure S1); therefore, plants were infiltrated with each recombinant at OD_600_ = 0.5 in all subsequent experiments. Expression of VP2 (123 kD) and VP7 (37 kD) as well as the VP7 trimer (135 kD) was demonstrated, the proteins being visualized as distinct bands of the correct expected molecular weight in SDS‐PAGE analyses of crude extracts. Apparent differences in gel loading are sometimes observed as a result of natural leaf‐to‐leaf and plant‐to‐plant total soluble protein (TSP) variation. Bands corresponding to VP3 (103 kD) and VP5 (57 kD) were not detected due to a peculiarity of the available antiserum, which has been shown to detect only VP2 and VP7. However, fully formed AHSV‐5 VLPs were imaged by TEM analysis of these crude extracts, indicating that all four capsid proteins were expressed and indeed had self‐assembled into complete particles (Figure 2c). As such, this is the first known report of AHSV VLPs being produced in plants.

### Density gradient ultracentrifugation is a suitable purification method for plant‐produced AHSV‐5 VLPs

To produce an AHS VLP preparation of sufficient purity and concentration for immunization of guinea pigs, several modifications were made to the small‐scale expression protocol. Firstly, the process was upscaled to infiltrate 24 whole plants with the recombinant constructs at an OD_600_ of 0.5 each. Secondly, AHSV VP7 is known to form trimers which aggregate into crystalline structures in the cytoplasm of infected cells (Burroughs *et al*., 1994), and there is evidence to suggest that these crystals impede VLP formation by sequestering available soluble VP7 trimers and preventing them from incorporating into the core particle (Bekker *et al*., 2017; Maree *et al*., 2016). Therefore, a mutated version of the VP7 gene containing seven amino acid substitutions near the 3′ end (Bekker, 2015) was also synthesized and cloned into pEAQ‐*HT* to yield pEAQ‐AHS5‐VP7_mu._ Co‐infiltration with *Agrobacterium* strains carrying the VP2, VP3 and VP5 recombinants together with the mutated construct as opposed to the wild‐type VP7 construct yielded an increased concentration of VLPs (Figure S2). Therefore, the mutated VP7 construct was used in all further experiments. Thirdly, a vacuum infiltrator was used to introduce the *Agrobacterium* suspension into the leaf intercellular spaces as this was much less labour intensive than syringe infiltration and resulted in more uniform infiltration of plant leaves.

**Figure 3:**
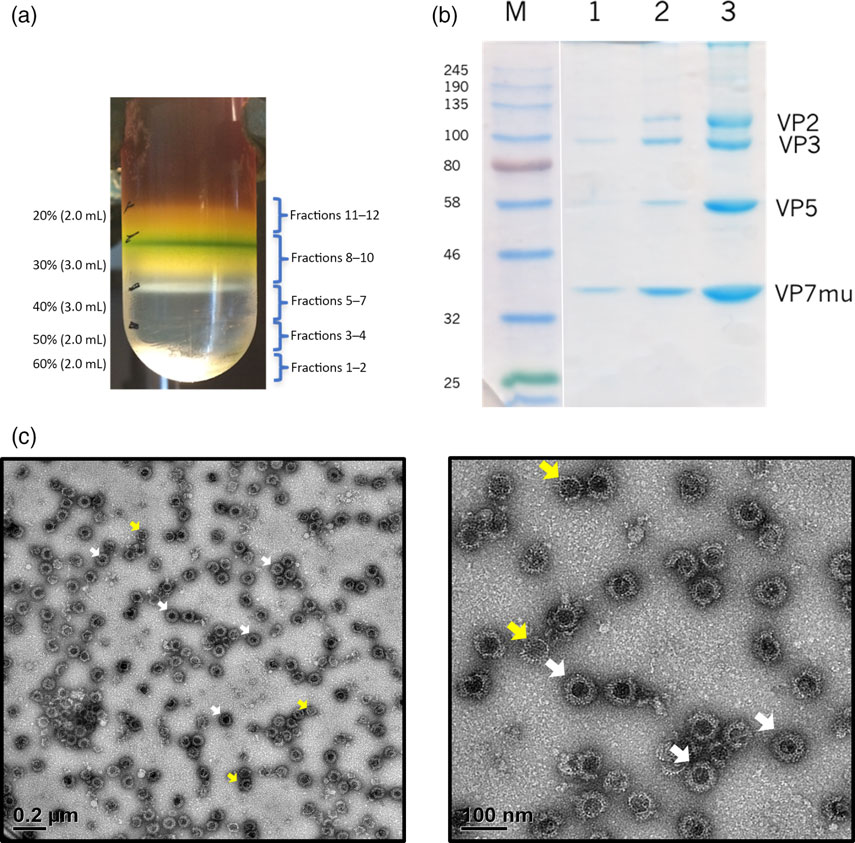
Purification of AHSV‐5 VLPs. (a) Six‐week‐old *N. benthamiana* plants were co‐infiltrated with all four AHSV‐5 *Agrobacterium* recombinants, leaves were harvested 7 days postinfiltration and the crude plant extracts were subjected to iodixanol density gradient ultracentrifugation. (b) Gradient fractions were collected from the bottom and all fractions separated by denaturing SDS‐PAGE followed by Coomassie blue staining. Fractions 6 (lane 1), 7 (lane 2) and 8 (lane 3) are shown with the location of the AHSV viral proteins VP2, VP3, VP5 and VP7_mu_ indicated to the right of the gel, while the molecular weight marker sizes are shown on the left. (c) Gradient fraction 8 was imaged by TEM revealing the presence of fully assembled VLPs (white arrows) together with some assembly intermediates (yellow arrows). Scale bars, 50–200 nm.

Clarified leaf extracts were purified by iodixanol density gradient ultracentrifugation. Green‐coloured impurities settled in the upper 30% region of the gradient, while a single iridescent band was observed at a higher density, near the 30%–40% interface (Figure 3a). Fractions were collected from the bottom of the tube and four distinct bands corresponding to the correct molecular weight sizes of the AHSV capsid proteins were observed following separation of fractions 6–8 by SDS‐PAGE and Coomassie blue staining (Figure 3b). These fractions also corresponded to the observed iridescent band. Expression of VP2 and VP7 as well as the VP7 trimer was demonstrated by Western blotting using the available AHSV‐5 antiserum (Figure S3). As AHS serotype 5 is one of the serotypes not included in the LAV, we did not have access to a positive control for this study. Therefore, the identity of the four protein species was further confirmed by mass spectrometry (Figure S4).

The co‐sedimentation of all four proteins was highly suggestive of the presence of VLPs, and this was confirmed by TEM analysis (Figure 3c). An estimated 40%–50% of the viral structures were seen to be complete AHSV VLPs (white arrows in Figure 3c) or contained at least a partial VP2 outer layer, although some particles appear to have been slightly damaged during the purification process. Assembly intermediates representing core‐like particles (CLPs) or CLPs in the process of acquiring the two outer coat proteins were also observed (yellow arrows in Figure 3c). Gel densitometry was used to estimate the VLP concentration (Figure S5). The purification was repeated several times and typically, 70 g infiltrated leaf material yielded ± 0.4 mg highly purified VLPs, which equates to ± 5.7 mg purified VLPs/kg leaf biomass.

### Plant‐produced AHSV‐5 VLPs induce a strong immunogenic response in guinea pigs

**Figure 4:**
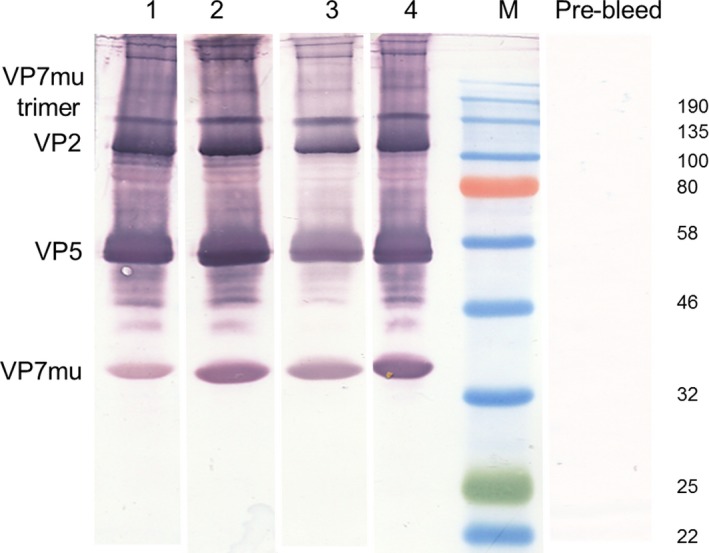
Immunogenicity of plant‐produced AHSV‐5 VLPs in guinea pigs. Vaccine and control guinea pig groups (*n* = 4) were vaccinated with plant‐produced AHSV‐5 VLPs (guinea pigs V2‐V5) or PBS (guinea pigs C2‐C5), respectively. Both vaccines were formulated with 5% Pet Gel A adjuvant (Seppic, Paris, France). Guinea pigs V2–V5 were immunized with a dose of 16.5 μg AHSV‐5 VLPs on day 0 and boosted with a dose of 50ug VLPs on day 13, while guinea pigs C2‐C5 were vaccinated with PBS as per the same schedule. Serum was collected on day 41 and antisera (1 : 10 000 dilution) from guinea pig V2 (lane 1), V3 (lane 2), V4 (lane 3) and V5 (lane 4) final bleeds were used to detect AHSV‐5 VLPs on standard Western blots separately. AHSV proteins were not detected by control guinea pig sera nor in any of the prebleed sera. The location of the AHSV viral proteins VP2, VP5 and VP7_mu_ as well as the VP7_mu_ trimer is indicated to the left of the blots, while the molecular weight marker sizes are shown on the right. No signal was detected for the innermost core protein VP3.

Guinea pigs were used as a small animal model to test the ability of the plant‐produced AHSV‐5 VLPs to induce an immune response. On day 0, five guinea pigs (V1–V5) were each vaccinated with AHSV VLPs mixed with 5% Pet Gel A adjuvant (Seppic, Paris, France), while five control animals (C1‐C5) were immunized with PBS plus adjuvant. Prior to the boost inoculation, a further purification was carried out which yielded sufficient AHS VLPs to increase the amount of the next inoculum. As this was a first vaccination study, the optimal dose required was unknown and animals were therefore boosted on day 13 with a greater amount of VLPs than used for the prime inoculation, or PBS. Due to accidental traumatic injury, guinea pig V1 was euthanized on day 15 and, together with control guinea pig C1, was excluded from the main study results. Sera from all other animals were collected on day 41 and final and prebleed sera (1 : 10 000) were used to probe a Western blot of VLPs used in the initial inoculations. Strong signals for VP2, VP5 and VP7 (both monomers and trimers) were detected by final bleed sera but not by the control guinea pig sera (C2–C5) nor the prebleed sera from all VLP‐vaccinated guinea pigs (Figure 4).

### Antiserum from guinea pigs immunized with plant‐produced AHSV VLPs neutralized live virus

To test the ability of the sera to neutralize live virus, serum samples from all guinea pigs were sent to the Equine Research Centre, Faculty of Veterinary Science, University of Pretoria, for serum neutralization tests. Sera were assayed against AHSV‐5 and AHSV‐8 as serological cross‐protection has been shown *in vitro* between serotypes 5 and 8 (von Teichman *et al*., 2010), and AHSV‐4 for which no cross‐protection has been shown. All VLP‐vaccinated guinea pig sera showed a high level of neutralization capability against AHSV‐5 and neutralized AHSV‐8 to a lesser extent, but to a similar degree compared to the AHS‐positive control (Table 1). The sera did not neutralize AHSV‐4 and control guinea pig sera did not neutralize any of the AHSV serotypes. These results indicate that plant‐produced AHSV‐5 VLPs stimulate a highly protective immune response in guinea pigs.

**Table 1:** Virus neutralizing antibody titres of serum samples from vaccinated (V) and control (C) guinea pigs. The guinea pig sera were assayed for neutralization capability against AHSV‐5, AHSV‐4 and AHSV‐8, as serological cross‐protection has been shown *in vitro* between serotypes 5 and 8, but not between serotypes 5 and 4. Horse serum from animals vaccinated with the AHSV live‐attenuated vaccine produced by Onderstepoort Biological Products (OBP) was used as a positive control

Group	Guinea pig	AHSV‐4	AHSV‐5	AHSV‐8
Plant vaccine	V2	Negative	1 : 5120	1 : 160
	V3	Negative	1 : 640	1 : 80
	V4	Negative	1 : 1280	1 : 56
	V5	Negative	1 : 2560	1 : 80
Control	C2	Negative	Negative	Negative
	C3	Negative	Negative	Negative
	C4	Negative	Negative	Negative
	C5	Negative	Negative	Negative
OBP vaccine	–	1 : 112	1 : 112	1 : 112

## Discussion

The results presented here show that the four AHSV 5 capsid proteins, VP2, VP3, VP5 and VP7_mu_, spontaneously assemble into virus‐like particles (VLPs), following recombinant DNA expression of these proteins within transiently transfected plant cells. The VLPs are noninfectious and inherently safe, as they assemble in the absence of any viral genetic material or other viral proteins. The proteins were each expressed from different recombinant‐carrying *Agrobacterium* strains co‐infiltrated in equal concentrations, and particles formed within 7 days. VLPs seen in crude extracts resulting from small‐scale expression in a single plant were almost all fully formed, whereas larger‐scale expression and subsequent iodixanol gradient purification produced a more heterogeneous mix of particles including assembly intermediates as well as some partially damaged particles. This phenomenon was observed regardless of whether particles contained the wild‐type or mutated VP7 protein, indicating that minor modification of VP7 by seven amino acid substitutions did not impact on the nature of viral particle formation. However, modifying VP7 in this way did appear to increase the number of particles produced relative to use of the wild‐type VP7. The average number of particles counted over 10 fields of view at a magnification of x54 000 was 9.2 for particles containing the wild‐type VP7 protein compared to 30.7 for particles incorporating the mutated VP7. Furthermore, a comparison of Western blot analyses of gradient fractions containing the two VLP species also seemed to indicate greater VLP formation with VP7_mu_ expression (Figure S2). VP2 was detected to a greater degree when VP7 was more available for incorporation into viral cores, implying increased particle formation. This is in agreement with work performed by others (Maree *et al*., 2016) who demonstrated an improvement in the efficiency of core‐like particle (CLP) production following co‐expression of VP3 with VP7 containing a 6‐mer peptide insertion mutation in a surface‐exposed loop located in the lower part of the top domain. Both their and our studies suggest that AHSV VLP formation can be enhanced by genetic modification of VP7 leading to increased availability of VP7 trimers for incorporation into the AHSV core particle.

Density gradient ultracentrifugation is a useful purification strategy for confirming the successful assembly of VLPs within the plants, but it is possible that the high centrifugal force may be damaging to the particles. Indeed, it has been our observation that crude plant extracts contain a much higher percentage of fully formed VLPs, albeit at a much lower concentration, compared to VLPs in iodixanol gradient fractions. This, together with the high cost of iodixanol, the expensive and sensitive equipment required and the labour‐intensive centrifugation and fractionation step, makes it important to consider alternative purification strategies. These could include depth filtration and tangential flow filtration for large‐scale centrifuge‐free production. However, although some of the purified particles appeared incomplete and thus probably did not contain the full complement of VP2, sufficient VP2 was present to elicit a strong serotype‐specific neutralizing antibody response when injected into guinea pigs.

The guinea pig has been shown by others to be a useful laboratory model for evaluating the antigenic properties of AHS vaccines (Erasmus, 1978; Lelli *et al*., 2013; Ronchi *et al*., 2012). We therefore tested the efficacy of our candidate vaccine in guinea pigs prior to testing it in horses, and have demonstrated that all the animals vaccinated with the plant‐produced AHSV‐5 VLPs seroconverted after two doses of the vaccine. Vaccination did not cause any adverse reaction in the guinea pigs which, aside from the guinea pig which was injured during handling and had to be euthanized, all remained healthy to the end‐point of the study.

The guinea pigs vaccinated in this study developed virus neutralizing antibody (VNAb) titres ranging between 640 and 5120 (2.8 and 3.7 log10) against homologous AHSV‐5 VLPs. These results exceed VNAb titres obtained in studies by others using poxvirus vectors expressing the outer capsid proteins VP2 and VP5 (Alberca *et al*., 2014; Guthrie *et al*., 2009). In the study by Guthrie *et al*. (2009), horses vaccinated with 10^7.1^ TCID50 of a canary pox‐based AHSV vaccine (ALVAC®‐AHSV) expressing VP2 and VP5 of AHSV‐4 developed serum VNAb titres of 20–40 (1.3–1.6 log10). Alberca *et al*. (2014) vaccinated horses with 10^8^ pfu/mL of a modified vaccinia Ankara (MVA) virus expressing VP2 of serotype 9 and reported VNAb titres of 1.6–2.4 log10. In this study, guinea pigs were first each vaccinated with 16.5 μg plant‐produced VLPs as this was the amount of highly purified VLPs available at the start of the study. Subsequent to the initial vaccination, further purifications yielded sufficient VLPs to increase this dosage. Because no previous studies using plant‐produced AHSV VLPs have been described and as Theunemann *et al*. showed that 50 μg plant‐produced BTV VLPs gave good protection against BTV in sheep, we decided to boost inoculate the guinea pigs with 50 μg VLPs instead of a second dose of 16.5 μg. The unfortunate accidental injury and subsequent euthanization of one of the guinea pigs (V1) 2 days after the boost vaccination (day 15) gave us the opportunity to test the immune response prior to the end of the study. Even at this early stage, sera from this guinea pig developed a VNAb titre of 224, twice that of the AHS‐positive control (112) (Table S1). It is therefore feasible to suggest that 50 μg was probably considerably in excess of the VLP dose required for guinea pigs, and could quite possibly be a sufficient dose for horses even though they are much larger animals.

Our results thus demonstrate that fully assembled AHSV‐5 VLPs could be produced in plants via a transient expression strategy and that plant‐produced AHSV 5 VLPs elicit a strong serotype‐specific neutralizing antibody response in guinea pigs. Our study also confirms the suitability of the guinea pig as a small animal model for preliminary testing of recombinant AHSV vaccines. The study will now be extended to include safety and immunogenicity testing in horses, the main target animals, with a view to producing a supplemental vaccine to AHSV‐5 to complement the standard live vaccine mixture.

## Experimental procedures

### Constructs

A consensus gene sequence for each of the AHSV‐5 viral capsid proteins VP2, VP3, VP5, and VP7 was obtained by aligning sequences (4–12 available) for these genes listed in GenBank, using CLC Mainbench bioinformatics software (Qiagen Bioinformatics, Aarhus, Denmark). Consensus sequences were codon‐optimized for expression in *N. benthamiana* and synthesized by GenScript Biotech Corporation (Nanjing, China) with flanking *AgeI* and *XhoI* restriction enzyme sites. The codon‐optimized *VP7* consensus sequence, modified as described by S. Bekker (2015) to include seven amino acid substitutions near the 3′ end (Pro_276_His, Arg_328_Ala, Val_333_Asn, Ala_334_Pro, Pro_335_Met, Val_336_Pro and Gln_338_Pro), was also synthesized. Restriction enzyme cloning was used to insert the genes into the pEAQ‐*HT* expression vector obtained from George Lomonossoff, John Innes Centre, UK (Sainsbury *et al*., 2009), to produce pEAQ‐AHS5‐VP2, pEAQ‐AHS5‐VP3, pEAQ‐AHS5‐VP5, pEAQ‐AHS5‐VP7 and pEAQ‐AHS5‐VP7_mu_. The AHSV‐5 plasmid constructs were electroporated into *Agrobacterium radiobacter AGL1‐ATCC BAA‐101* as described previously (Maclean *et al*., 2007), and recombinant clones were selected at 27 °C on Luria–Bertani (LB) media plates containing 25 μg/mL carbenicillin and 50 μg/mL kanamycin.

### Transient expression in plants

Expression of the AHSV‐5 capsid proteins was achieved by agroinfiltration of 5‐ to 6‐week‐old *N. benthamiana* plants. *Agrobacterium* transformants each carrying one of the AHSV‐5 capsid protein genes were subcultured and grown overnight with agitation at 27 °C in Luria–Bertani broth (LBB) base supplemented with 50 μg/mL kanamycin, 20 μm acetosyringone and 2 mm MgSO_4_. The cultures were diluted in resuspension solution (10 mm MES, pH 5.6, 10 mm MgCl_2_, 100 μm acetosyringone) to the desired optical density and incubated for 1 h at 22 °C to allow for expression of the *vir* genes. For single infiltrations, each AHSV‐5 *Agrobacterium* recombinant suspension was diluted to OD_600_ = 0.5 or 1.0, while co‐infiltration suspensions contained all four AHSV‐5 recombinants in a ratio VP2 : VP3 : VP5 : VP7 of 1 : 1 : 1 : 1 or 1 : 1 : 2 : 1. Plants were grown at 22–25 °C under 16‐h/8‐h light/dark cycles. *Agrobacterium* suspensions were infiltrated into the leaf intercellular spaces using either a blunt‐ended syringe or a vacuum infiltrator, applying a vacuum of 100 kPa. For optimization of the expression, three leaf discs were obtained from each plant, clipped with the lid of a microcentrifuge tube on 3, 5 and 7 days postinfiltration (dpi) and homogenized in three volumes of PI buffer [phosphate‐buffered saline (PBS), pH 7.4, containing 1× Complete protease inhibitor cocktail (Roche, Basel, Switzerland)] using a micropestle. The homogenate was incubated on ice for 30 min and then clarified by centrifugation at 13 000 rpm for 15 min in a benchtop microfuge. SDS‐PAGE samples were prepared by adding 50 μL 5× sample application buffer to 200 μL clarified crude extract and heat‐denatured at 95 °C for 5 min before loading 40 μL per gel lane. For large‐scale expression, leaf tissue was harvested 7 dpi, as this time span was shown to be optimal for expression of all four capsid proteins. Harvested leaves were immediately homogenized in three volumes PI buffer using a Moulinex™ juice extractor. The homogenized leaves were re‐incubated with the extracted juice and incubated at 4 °C for 1 h with gentle shaking. Crude plant extracts were filtered through four layers of Miracloth™ (Merck, Darmstadt, Germany) and the filtrate was clarified by centrifugation at 13 000 rpm for 15 min at 4 °C.

### Purification and Western blot analysis

AHSV‐5 VLPs were purified by iodixanol density gradient ultracentrifugation. Iodixanol (Optiprep™; Sigma Aldrich, St Louis, MO) solutions (20%–60%), prepared in PBS, were used to create a 12 mL step gradient (2–3 mL of each gradient in 10% incrementing steps) under 27 mL clarified plant extract and centrifuged at 32 000 rpm for 2 h at 4 °C in an SW 32 Ti rotor (Beckman, Brea, CA). Fractions of 1 mL were collected from the bottom of the tube and 30 μL from fractions representing the 30%–40% region of the gradient was electrophoresed on a 10% SDS‐polyacrylamide gel, followed by Coomassie blue staining. Particle quantification was achieved by visual comparison of the four capsid protein bands to known amounts of bovine serum albumin (BSA) run in separate lanes on the same SDS‐PAGE gel. To further purify and concentrate VLP samples for use in animal studies, VLP‐containing fractions were diluted with PBS to 20% iodixanol and subjected to a second round of ultracentrifugation as per the same protocol described above. Both crude plant extracts and gradient‐purified VLPs were analysed by Western blot: heat‐denatured samples were separated on 10% polyacrylamide gels and then transferred onto HyBond™ C Extra nitrocellulose membranes (AEC‐Amersham, Gauteng, South Africa) using a Trans‐blot® SD semi‐dry transfer cell (Bio‐Rad, Irvine, CA). Membranes were first probed with a 1 : 1000 dilution of AHSV‐5‐specific horse serum (received from Dr C. Potgieter, Deltamune, Pretoria, South Africa), washed four times with PBS containing 0.05% Tween® 20 (Sigma Aldrich, St Louis, MO) (PBS‐T) and then probed with 1 : 5000 dilution of anti‐horse alkaline phosphatase‐conjugated secondary antibody (Sigma Aldrich, St Louis, Missouri). After washing again, proteins were detected with 5‐broo‐4‐chloro‐3‐indoxyl‐phosphate (BCIP) and nitroblue tetrazolium (NBT) phosphatase substrate (BCIP/NBT 1‐component, KPL, SeraCare, Milford, MA).

### Mass spectrometry

The identities of the protein species observed on the Coomassie‐stained gel were independently determined by the Centre for Proteomic and Genomic Research (CPGR, Cape Town, South Africa). Gel pieces were washed and fragmented by in‐gel trypsin digestion as per the protocol described by Shevchenko *et al*. (2007). The peptide solution was analysed using a Dionex Ultimate 3000 nano‐HPLC system (ThermoFischer Scientific, Waltham, MA) coupled to a Q Exactive™ Hybrid Quadrupole‐Orbitrap Mass Spectrometer (ThermoFischer Scientific). Byonic software (Protein Metrics, San Carlos) was used for comparison of the spectra with sequences retrieved from the UniProt Swissprot protein database. Samples were interrogated against *Nicotiana* spp*, Agrobacterium* spp and African horse sickness virus proteomes.

### Transmission electron microscopy

Glow‐discharged copper grids (mesh size 200) were floated on 20 μL crude plant extract or 20 μL density gradient fractions for 3 min and then washed successively by floating on five drops of sterile water. Particles were negatively stained for 30 seconds with 2% uranyl acetate and then imaged using a Technai G2 transmission electron microscope (TEM).

### Immunization of guinea pigs

Approval for the immunization experiments was obtained from the Faculty of Health Sciences Animal Ethics Committee, University of Cape Town (FHS AEC ref No.: 016/019). Prior to the study, 100 μL of blood was drawn from each of 10 female guinea pigs (Hartley strain). Guinea pigs (*n* = 5) were injected subcutaneously with 16.5 μg purified AHSV‐5 VLPs or 30% iodixanol in PBS, both formulated in 5% Montanide PET Gel A adjuvant (Seppic, Paris, France). Animals were boosted once on day 13 with 50 μg VLPs or PBS, and on day 41, they were euthanized by anaesthesia with ketamine/xylazine and exsanguinated. Serum was tested for antibodies by Western blot analysis, where guinea pig antisera were used at a dilution of 1 : 10 000 as per the protocol described above.

### Neutralization assays

The serum neutralizing antibody titres of individual guinea pig sera were assayed against three different AHSV serotypes, namely serotypes 4, 5 and 8 using a serum neutralization test (SNT) as previously described (House *et al*., 1990). This was carried out by Ms Carina Lourens at the Equine Research Centre, Faculty of Veterinary Science, University of Pretoria.

## Supporting information


**Figure S1** Optimization of plant‐based expression of recombinant AHSV‐5 structural proteins.

**Figure S2** Increased formation of AHSV‐5 VLPs incorporating a mutated version of VP7.

**Figure S3** Purification of AHSV‐5 VLPs.

**Figure S4** Mass spectrometry analysis of the 4 protein bands recovered from SDS‐PAGE separation of density gradient fractions from leaves co‐infiltrated with *Agrobacterium* AGL1 pEAQ recombinants for co‐expression of AHSV capsid proteins VP2, VP3, VP5 and VP7_mu_.

**Figure S5** Quantification of AHSV‐5 VLPs by gel densitometry.

**Table S1** Virus neutralizing antibody titres of serum samples from vaccinated and control guinea pigs V1 and C1.
