# Supplementary figures and images for: Immunogenicity of plant‐produced African horse sickness virus‐like particles: implications for a novel vaccine
Source: Plant Biotechnol J. 2017 Aug 1;16(2):442–50. doi: 10.1111/pbi.12783 (PMC5787833; doi:10.1111/pbi.12783)

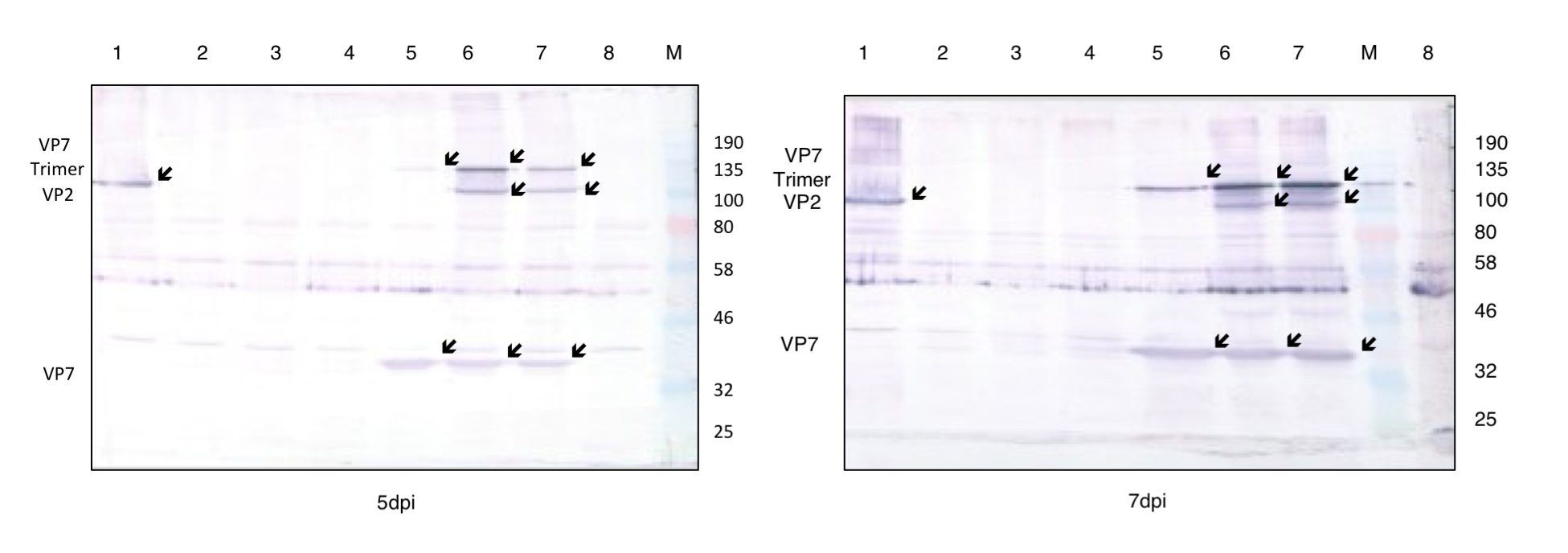

Supplement: Supplementary file 1 — Figure S1 Optimization of plant‐based expression of recombinant AHSV‐5 structural proteins. [file PBI-16-442-s005.tiff]

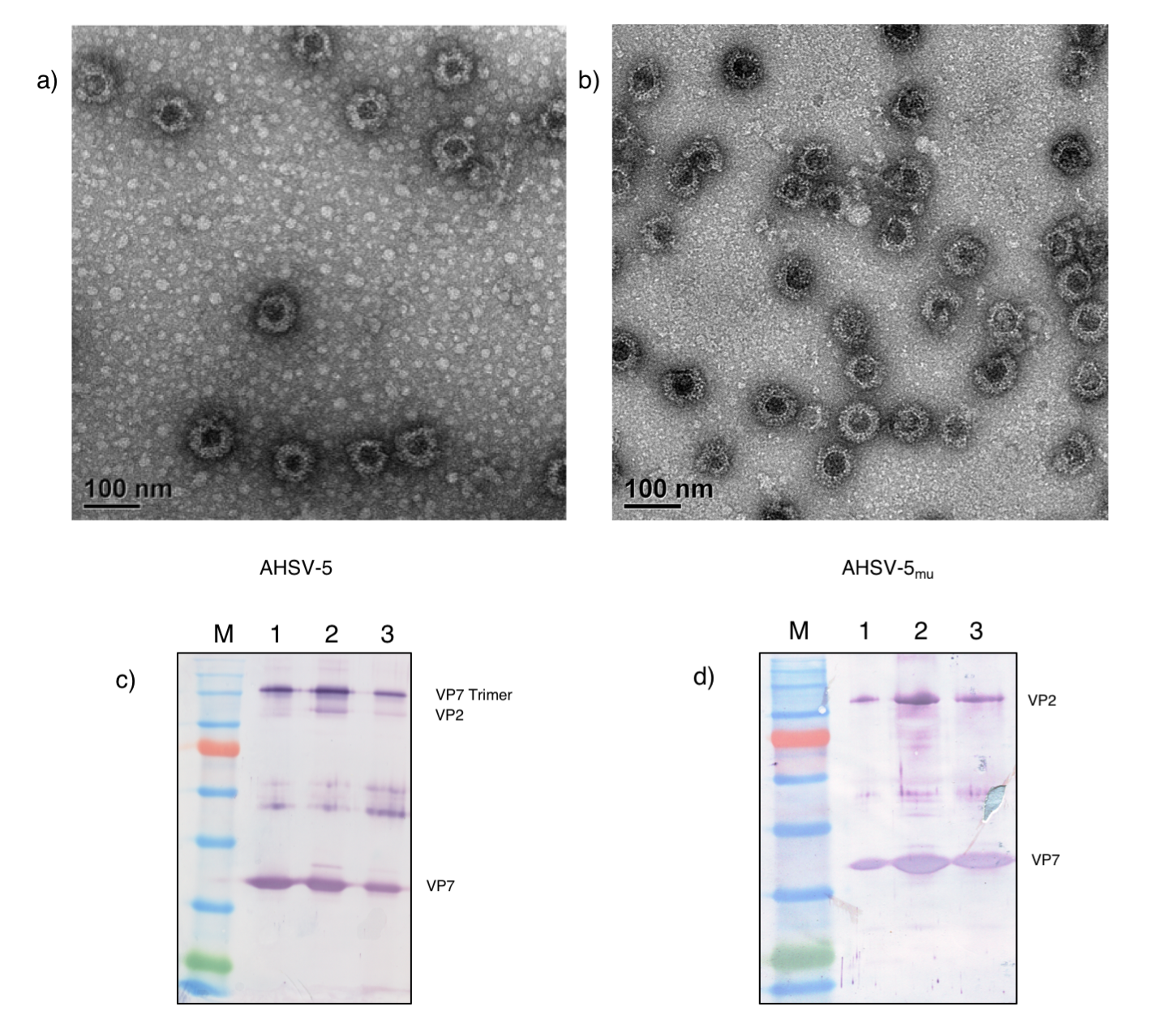

Supplement: Supplementary file 2 — Figure S2 Increased formation of AHSV‐5 VLPs incorporating a mutated version of VP7. [file PBI-16-442-s004.tif]

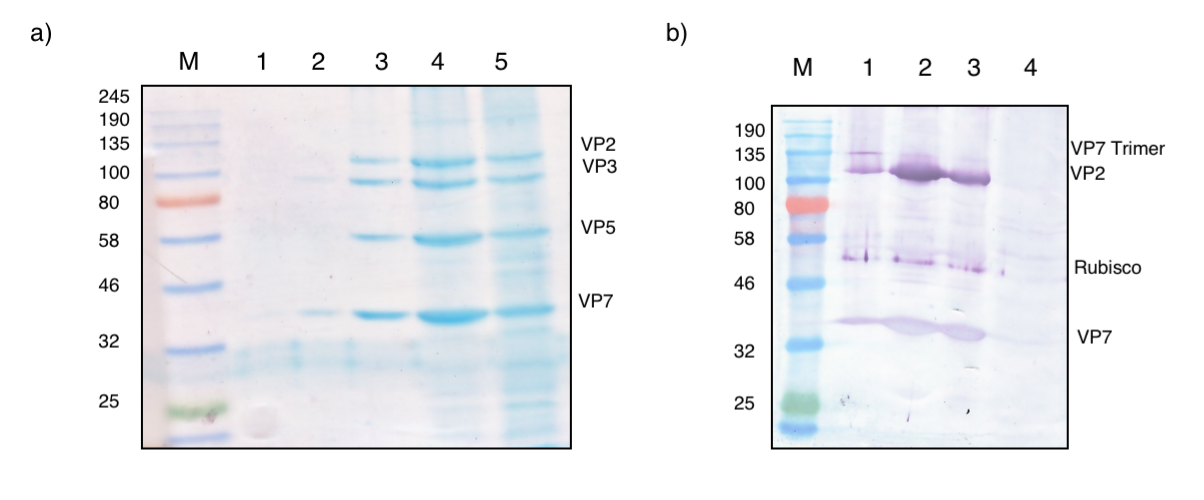

Supplement: Supplementary file 3 — Figure S3 Purification of AHSV‐5 VLPs. [file PBI-16-442-s003.tif]

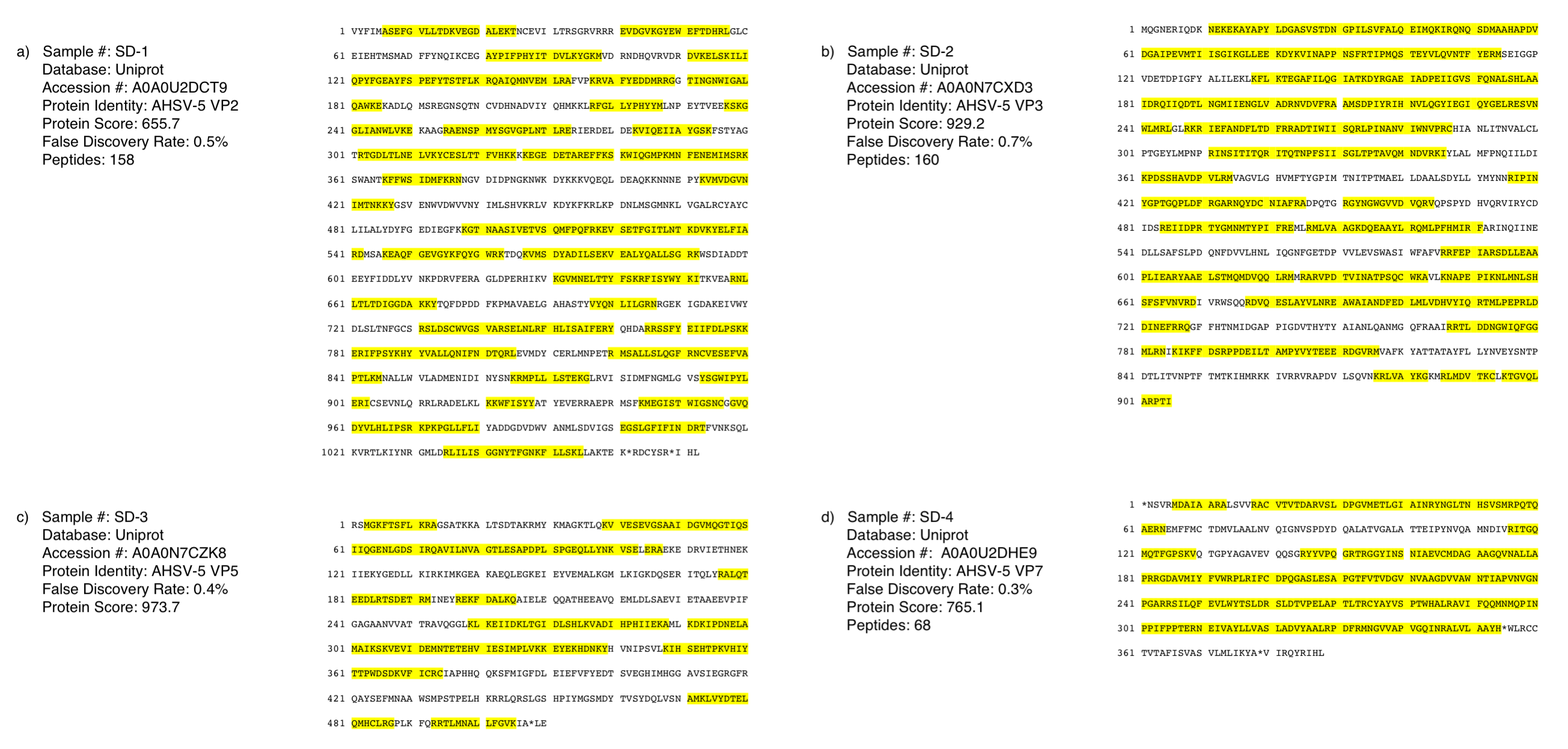

Supplement: Supplementary file 4 — Figure S4 Mass spectrometry analysis of the 4 protein bands recovered from SDS‐PAGE separation of density gradient fractions from leaves co‐infiltrated with Agrobacterium AGL1 pEAQ recombinants for co‐expression of AHSV capsid proteins VP2, VP3, VP5 and VP7mu. [file PBI-16-442-s002.tiff]

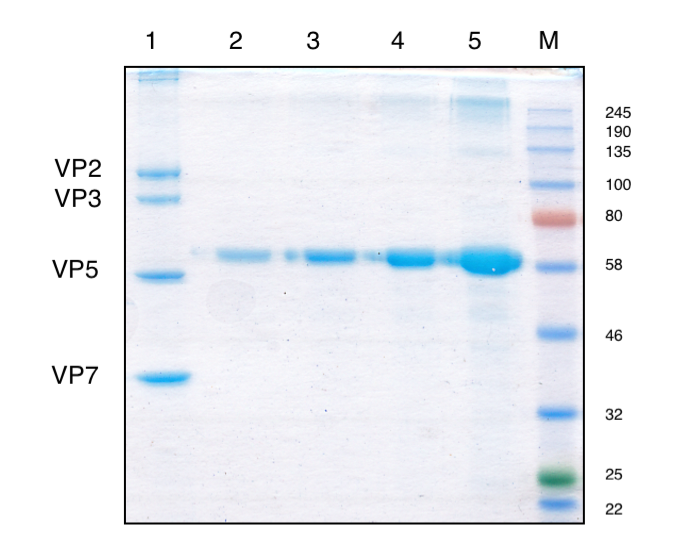

Supplement: Supplementary file 5 — Figure S5 Quantification of AHSV‐5 VLPs by gel densitometry. [file PBI-16-442-s006.tiff]
