# Supplementary material for: Immunogenicity of plant‐produced African horse sickness virus‐like particles: implications for a novel vaccine
Source: Plant Biotechnol J. 2017 Aug 1;16(2):442–50. doi: 10.1111/pbi.12783 (PMC5787833; doi:10.1111/pbi.12783)
Supplement: Supplementary file 6 — Table S1 Virus neutralizing antibody titres of serum samples from vaccinated and control guinea pigs V1 and C1. [file PBI-16-442-s001.docx]

**Table S1**

Group Guinea pig AHSV-4 AHSV-5 AHSV-8

Plant vaccine V1 Negative 1:224 1:20

Control C2 Negative Negative Negative

OBP vaccine - 1:112 1:112 1:112

______________________________________________________________________
